# A Comprehensive Focus on Global Spectrum of *BRCA1* and *BRCA2* Mutations in Breast Cancer

**DOI:** 10.1155/2013/928562

**Published:** 2013-11-07

**Authors:** Fatemeh Karami, Parvin Mehdipour

**Affiliations:** Department of Medical Genetics, Tehran University of Medical Sciences, School of Medicine, Tehran, Iran

## Abstract

Breast cancer (BC) is the most common cancer of women all over the world. *BRCA1* and *BRCA2* gene mutations comprise the most important genetic susceptibility of BC. Except for few common mutations, the spectrum of *BRCA1* and *BRCA2* mutations is heterogeneous in diverse populations. 185AGdel and 5382insC are the most important *BRCA1* and *BRCA2* alterations which have been encountered in most of the populations. After those Ashkenazi founder mutations, 300T>G also demonstrated sparse frequency in African American and European populations. This review affords quick access to the most frequent alterations among various populations which could be helpful in BRCA screening programs.

## 1. Introduction

Breast cancer (BC) is the most common malignancy in females in almost all of countries with highest age-adjusted incidence in developed countries (73%) and includes the 23% of all types of cancers. World Health Organization (WHO) report shows that this incidence increases 2% per year [1]. The incidence rate in Iranian women is found to be 16.2 per 100,000 people per year which is standardized for age, and the mortality rate is 5.5 per 100,000 people per year [2]. As a matter of fact, BC is responsible for most of deaths due to cancer in women all over the world. According to surveys which were globally conducted, the overall rate of BC is higher in American and European women compared to the Asian, and it may be related to the life style of Asian population [3]. The major type of BC is found to be sporadic with estimated frequency ranging from 90% to 95%, and the rest (5–10%) includes the familial BC [4]. It was demonstrated that BC patients in Iran are usually younger than European or American counterparts given that familial or hereditary BC comprises the main risk factor among Iranian woman. 

 The most important genes which were proposed for inherited BC as high penetrant genetic susceptibility include *BRCA1* and *BRCA2*, *p53*, *PTEN*, *STK11/LKB1*, and *CDH1* genes [3], while some others like *ATM, CHECK2, BRIP1, *and *PALB2* are considered as moderate genetic factors. *BRCA1* and *BRCA2* genes will be discussed in detail in the following sections of this paper.

*P53* is a known gene involved in Li-Fraumeni cancer syndrome and could be identified in 20%–35% of breast tumors and has about 1400 mutation types which almost involve transactivation domain. 

BC shows 4% more risk in women who are carrier of *PTEN* gene mutations. This gene encodes the phosphatase and tensin homologue and its mutations is often found in ductal carcinoma and triple negative forms of BC. Promoter hypermethylation has been suggested as a probable mechanism of loss of heterozygosity (LOH) in breast tumor [4]. It was described that *PTEN* loss of function not only is involved in tumor formation but also causes resistance to targeted therapy. 

Studies could not find somatic mutation in *STK11/LKB1* locus on chromosome 19p which is responsible for Peutz-Jeghers syndrome (PJS) and only the LOH of this tumor suppressor locus was determined in BC samples. It is taught that the aforementioned locus is accompanied by 5 times more risk of BC in PJS patients [5].

*CDH1* gene encodes for E-cadherin which is determined as a primary indicator in addition to estrogen receptor *α* (ER*α*) for luminal epithelial tumors of breast [5]. Women who are carriers of *CDH1* mutations have 39%–52% to be affected with BC in their life. It was identified that most of the nonsynonymous mutations have impact on three-dimensional structure of this protein [6]. On the other hand, *CDH1* underexpression is associated with more metastasis and poor prognosis in either ER positive or negative BCs [7].

Ataxia telangiectasia-mutated (*ATM*) gene encodes for ATM protein which is involved in double-stranded breaks DNA repair and regulation of cell cycle. It was shown that being carrier of heterozygote mutations of this gene is associated with increased risk of BC. We have demonstrated the D1853N polymorphism in exon 36 of *ATM* gene is significantly more frequent in BC patients compared to the external and internal control groups [8]. Based on our proposed three-hit hypothesis, D1853N in conjunction of IVS 38- 63T>A and IVS 38- 30 A>G within *ATM* gene, comprised the triangle of astrocytoma development in an Iranian proband affected with astrocytoma as well [8, 9].

The greater risk belongs to *BRCA1* and *BRCA2 *genes which enhance the risk of BC progression up to 59%–87% and 38%–80% respectively. *BRCA1* gene mutations accounts for 1-2% BC and approximately all of the familial breast-ovary tumors. It seems that *BRCA1* and *BRCA2* gene mutations are associated with higher grades of breast tumors [10]. BRCA proteins are involved in repair of break in double-strand DNA through homologous recombination and also cell cycle progression. Loss of function in *BRCA1* and *BRCA2* genes is responsible for male BC [10]. The mutations in both proteins also increase the risk of ovarian and prostate cancers with special risk of pancreatic cancer for *BRCA2* and colon cancer for *BRCA1* mutations [11]. The *BRCA1* and *BRCA2* defective tumors demonstrate specific pattern of genetic alterations required for tumor genesis; for example, *Myb* amplification and *p53* mutation with different pattern frequently occur in *BRCA1* deficient tumors compared to sporadic ones; this is in contrast to *Her2* and *cyclin D1* overexpression. In addition, we could often detect the *BRCA1* mutations in ER and PR negative BCs, so they have poor response to tamoxifen therapy [12, 13].

## 2. Structure of *BRCA1* and *BRCA2* Genes and Proteins 

The BC susceptibility gene, *BRCA1*, is located at chromosome 17q21 and codes for an 1863 amino acid protein involved in gene regulation processes following DNA damage. *BRCA2* gene was mapped on chromosome 13q12.3 by positional cloning and translation following its transcription which leads to the formation of a protein with 3,418 amino acids. About 41.5% of *BRCA1* gene is unusually composed of Alu sequences versus low density of other repeat sequences (4.8%) [14].

*BRCA1* and *BRCA2* genes have 24 and 27 exons, respectively. The largest exon in both genes is exon 11 (3.5 kbp in *BRCA1*) which harbors the most important and frequent mutations in BC patients. In contrast, one of the noncoding regions of genome is located in exon 1 of both *BRCA1 *and *BRCA2 *genes.

**Figure 1:**
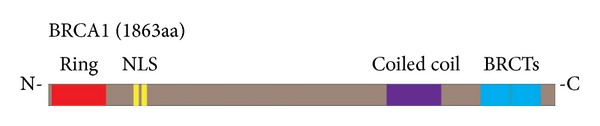
*BRCA1* protein structure with different domains [15].

The overall structure of *BRCA1* protein comprises of zinc finger in amino terminal, C3HC4 type (RING finger) which creates a loop structure through binding two zinc atoms by eight amino acids. In addition, the *BRCA1* C Terminus (BRCT) domain, nuclear localization signal, and nuclear export signal motifs could be found within *BRCA1* protein structure (Figure 1). 

**Figure 2:**
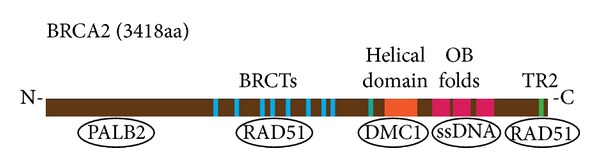
*BRCA2* protein structure with different binding sites [15].

All the proteins containing RING finger have role in ubiquitination pathway as E3 ubiquitin ligase. The Zinc finger structure contains Cys3HisCys4 amino acid motif which is highly conserved and every change in this amino acid composition could affect binding properties of *BRCA1* protein. *BRCA1* is involved in transcriptional regulation through interaction with histone deacetylase complexes via its BRCT domain. The similar BRCT domain called BRC motif is found in proteins as well as *BRCA2* which are participated in cell cycle checkpoint and function in response to DNA damage. It is associated with the area for single-strand binding and a region with 26 amino acids called PhePP motif that binds to DMC1 which is a meiosis-specific recombinase. A domain binding single-stranded DNA containing 3 oligonucleotide-binding (OB1-3) folds and a helix-turn-helix motif that binds double stranded DNA is located in C-terminal segment of *BRCA2*. Moreover, there is a region in the 3′ end of *BRCA2* gene, in exon 11, called ovarian cancer cluster region (OCCR) which may increases the risk of ovarian cancer in *BRCA2* families if become mutated [16, 17].  Figure 2 shows the different interaction sites of *BRCA2*.

*BRCA1* combines with other tumor suppressors, DNA damage sensors, and signal transducers to form a large multisubunit protein complex known as the *BRCA1*-associated genome surveillance complex (BASC).

*BRCA1* and *BRCA2* orthologs have been identified in most mammals for which complete genome data are available [18].

## 3. *BRCA1* and *BRCA2* Mutations

Over 2000 different mutations have been reported in *BRCA1/2* genes including deletions, insertions, and many single nucleotide substitutions in coding or noncoding sequences. The most common types of mutation are attributed to small insertion/deletion frameshift, nonsynonymous truncation, and disruption of splice site leading to entire nonfunctional BRCA proteins. The higher rate of duplication/deletion in *BRCA1* gene versus *BRCA2* (42% and 20%, resp.) is due to accumulation of Alu sequences [19]. Large genomic rearrangements (LGRs) comprise about 1/3 of all mutations occurring in *BRCA1* gene which are typically a result of homologous recombination between *BRCA1* gene and the same pseudogene sequences [14].

In *BRCA2* gene, most of mutations occur in exons 10 and 11 and usually include insertions or deletions which raise the missense alterations and premature stop codon ending in truncated and nonfunctional protein. It was shown that the segments of *BRCA2* that were separated out contain double strand break domain (DBD), nuclear localization signal (NLS), and Rad-51 binding motif are located in C-terminal which are critical for *BRCA2* function [20].

All of the most important and frequent *BRCA1* and *BRCA2* mutations which have been discovered so far are represented in Tables 2, 3, 4, 5, 6, 7, and 8. Some of the *BRCA1/2* mutations show population specific pattern and some of them have been found in various studies from different populations.

In this review we are intended to describe the most important polymorphisms at a glance and then the most frequent and important mutations of different populations from all over the world based on the studies which have been freely published online up to April 2013. Finally, we will discuss about the genotype-phenotype correlation of *BRCA1/2* gene alterations highlighted in various ethnicity.

## 4. Polymorphisms and Variants in *BRCA1* and *BRCA2* Genes at a Glance

There are many reports for different polymorphisms within *BRCA1* and *BRCA2* genes all over the world. They are classified into four major categories: silent, nonsynonymous, harmful, and unclassified whose impacts on *BRCA1/2* genes remain to be clear. In this section, we will take a look at the most important variants discovered in different populations whose promoting or protective role in BC development has been verified (Table 1).

S1613G in exon 16 and P871L accompanying E1038G in exon 11 constitute the most common single nucleotide polymorphisms in *BRCA1* gene which were frequently reported in BC family assays of India, Greek, Malay, Sri Lanka, Turkey, and Italy. S1613G is responsible for amino acid switch from serine to glycine which was detected among Italian women who were carriers of the major *BRCA1* mutations [21–25]. Other polymorphisms in this gene are E879E, S919P, and Y1137Y. They have been replicated in different populations including Chinese and Finnish. Although the S919P takes place in BACH1 domain, it was not considered as a high predisposal factor of BC [26].

Among other variants which have been identified in *BRCA1*, c.1984C.T changes the histidine to tyrosine and disrupts the *BRCA1* protein function. So it could be also regarded as a pathogenic variant which was only detected in Cypriot population [27].

There are also some other variants in *BRCA1* which are associated with anomalous splicing and lead to premature translation and truncated protein including c.302-3C>G in *BRCA1* and c.475G>A in addition to c.7007G>A in *BRCA2*. They have been described in a Czech population assay and were considered as deleterious variants [28]. In addition, Y179C in exon 8 of *BRCA1* converting a conserved tyrosine was found in several German BC families along with Ashkenazi Jews [29], one Australian family [30], and an Italian family [31]. Functional study of *BRCA1* protein containing this and some other variants (Y105C, P142H, and E143 K) has identified that proline residue interferes with recognizing double strand break site in DNA and may eventually disturb the participation of *BRCA1* protein in DNA repair [32]. Moreover, C5242A (A1708E) polymorphism in exon 18 of *BRCA1* gene leads to exchange of alanine to glutamine residues. owing to its occurrence in BRCT domain, it may interrupt the interaction of *BRCA1* protein with others especially those being involved in DNA repair. It was discovered in Hispanic BC families from Spain and El Salvador but it substantially comes from western European descent. It is maybe helpful to consider this variant in high risk Hispanic populations [33].

Furthermore, there is an insertion/deletion mutation in intron 24 (3′ UTR) of *BRCA1* gene that belongs to unclassified variants with unknown effect and was found in one of our study families with five BC patients [34]. 

There are three variants in *BRCA1* gene whose their protective effect against BC has been proposed. Since the lack of K1183R variant in exon 11 of *BRCA1* gene in which the two basic amino acids transform to each other (lysine to arginine) increases the risk of BC, it could be considered as another protective polymorphism. The second is RR genotype for Q356R polymorphism which has shown meaningful lower frequency in BC cases relative to controls [21]. Since, the intron variant, IVS7-34T.C has shown 40% more frequency in controls, a defending mechanism could be defined for it against BC in further studies [35, 36]. 

It was discovered that the 3232A>G base pair exchange was more seen versus 1342A>C mutation in carriers of *BRCA1* harmful alterations [37].

The association of S1832P, T2766I, N2781I, and K2860T polymorphisms in *BRCA2* with BC risk was defined in Danish population and it was predicted that they can negatively affect *BRCA2* function [38]. Moreover, c.9023A/C introduces a turn in *BRCA2* protein structure by replacing the proline with histidine. So this nonsynonymous variant may interfere with normal *BRCA2* function in Chilean families [39].

The c.72A>T (Leu24Phe) has been found in Finnish BC women recently. In silico assessments predicted that it possibly influences the *BRCA2* protein in pathogenic ways [3]. Although Miramar et al. described that some variants such as K3083E or 9475A>G introduce a drastic amino acid exchange (lysine to glutamine) and represent functional disturbance in *BRCA2* protein [40], they are still known as a nonpathogenic polymorphism. 4817A>G in exon 11 of *BRCA2* gene provides the suitable codon for converting the lysine residue to argenine and was only characterized in Romanian BC study. Given its pathologic effect on *BRCA2* protein function, it is regarded as a deleterious polymorphism in screening programs of this population [41].

Healy et al. have reported that a specific *BRCA2* variant (N372H) was associated with increased reproductive fitness in males in the United Kingdom and an increased BC risk in females. Based on the Meta-analysis review on the association of it with BC, it seems that 372H is not meaningfully associated with higher risk of BC in Asian, Caucasian, and African populations. However, further assays including controls samples are required to validate this result [42].

## 5. Global Distribution of *BRCA1* and *BRCA2* Mutations

### 5.1. Ashkenazi Jews

Ashkenazi Jewish population is one of the well-known population in which the founder mutations including 5382insC, 185delAG in *BRCA1*, and 6174delT in *BRCA2* were detected. These three mutations were also identified in other populations which will be discussed in the following sections. In a study on Ashkenazi Jews residing in USA, the *BRCA2* 3036del4 mutation was identified in only one BC patient diagnosed <50 yrs and the remainders were positive for the mentioned founder alterations [43]. However, 5382insC founder mutation was not found in Ashkenazi Jews from Brazil maybe due to selection of BC patients without familial history of BC [44].

### 5.2. Northern Europe

All the mutations regarding Northern European population were brought in Table 2.

In Finnish BC patients, *BRCA2* mutations have been reported more than *BRCA1* gene. The novel c.72A>T as destabilizing mutation, c.68-80insT, and c.793 + 34T>G alterations were identified. Except of a large deletion covering the exons 1–13 which were recognized in a family with ovarian cancer, no deletion/duplication has been detected in this population thus far [45]. Moreover, the 4088insA in *BRCA2* gene is found to be associated with a better prognosis of Finnish BC women [46].

Amongst Swedish population affected with BC, two founder mutations in *BRCA1* gene including 3172ins5 and 2594delC have been originated from Central Europe. The 1806C>T and 1201del11 alterations comprise the remainder of founders that were replicated in two Swedish assays. It is noteworthy that all of the *BRCA1* main alterations are mapped on exon 11. In addition, 4486delG is the most frequent and founder alteration in exon 11 of *BRCA2* gene [47, 48].

The major point of *BRCA1* and *BRCA2* screening in Danish population is that the prevalence of *BRCA2* mutations was found to be more frequent in west than in east Denmark. The most important and common alterations of *BRCA1* gene include: 2594delC, 5382insC, 3829delT, 3438G>T, 1675delA, and Q563X and for *BRCA2* 6601delA, 1538del4, 6714del4, and 999del5. Danish population shares some founder mutations with Norwegian and Swedish peoples that could propose a unique ancestry for these three Scandinavian countries [38, 49].

The most frequent mutations of *BRCA1* gene which are replicated in multiple Norwegian studies are 1135insA, 1675delA, 816delGT, 3203del11, and 3347delAG [50, 51]. The first two alterations are founder mutations of *BRCA1* gene in Norwegian population and it has been shown that they have less penetrance in them compared to Ashkenazi Jews [52, 53].

In Icelandic BC women, it seems that the rate of *BRCA1* mutations is relatively low. The only mutation detected was splice site alteration in exon 17 (Table 2). The only studied *BRCA2* mutation, 999del5, is well-known founder mutation of this population which is responsible for 24% of all BC patients who are <40 yrs and was identified in 40% of familial Icelandic BC [54–57].

*BRCA1* mutations do not affect risk of bilateral breast tumors in Dutch high risk individuals. Two frame shift deletions (del3835, 2804delAA) and the 1411insT single base insertion mutation have been recognized as founder alterations of Netherland. Furthermore, the 2804delAA deletion occurs in Alu repeat that disrupts the exon 13 and exon 22 sequences [58, 59].

### 5.3. Central and Southern Europe

All the mutations regarding Central and southern European population were brought in Table 3. In an Estonia population study, the well-known c.4154delA and c.5382insC rearrangements were found in 63.6% and 27% of all alterations, respectively. In addition, two novel frame shift deletions (c.6631delTTAAATG and c.3881-3882delGA) were recognized in exon 11 of *BRCA1 *gene which cause too early termination of translation. The c.4158A>G, c.4427T>C polymorphisms, similar to the previous studies showed no clinical adverse effects on *BRCA1* protein structure and function [60]. 

The c.3228_3229delAG and c.3285delA deletions in *BRCA1* gene are the most important and prevalent mutations of Italian populations (57%). The latter was found initially in Italian studies but the former mutation had high frequency only in male BC cases which may propose a mechanism for involvement of this deletion type in male BC pathogenesis. Once more, the founder alterations, c.1377_1378insA and c.5062_5064delTGT (72% of all *BRCA1* variants), were identified in *BRCA1* gene. Truncated *BRCA2* protein is created as a result of c.289G>T, c.2950G>T, c.7963C>T, and c.8878C>T mutations generating premature stop codon, and except of the last one, all of them were primarily detected in Italy. Screening for LGRs is important in Italian high risk families as they compose 19% of all the alterations usually affecting the *BRCA1* gene [61, 62]. Two new premature stop codon insertion/deletion mutations (7525_7526insT and 6174delT) were detected in exon 15 of *BRCA2* in Italian BC women. They occur in C-terminal of protein and are associated with disruption of nuclear localization signals (NLSs) which interfere with *BRCA2* nuclear penetration and participation in DNA repair process [20]. The significance of these alterations is due to their important role in drug resistance especially PARP inhibitors [63]. The *BRCA1* and *BRCA2* mutations screening program in Italian population was extended in BC patients from Apulia where two major missense polymorphisms, including S1613G (exon 16) and K1183R (exon11), were identified in *BRCA1* gene contributing to BC risk [21].

Spanish studies could discover two major founder mutations with low frequency in both *BRCA1/2* genes, the 330A>G substitution change in splice site between exon 5 and exon 6 which makes an inefficient truncated form of *BRCA1* protein [64]. The second change, 9254del5 in *BRCA2* gene, was also detected in French families [65]. However, the recent Spanish study introduced exons 3–5 del (g.8097_22733del14637) within the *BRCA1* gene as as a new primarily identified Spanish founder mutation among Valencian women [66]. The most frequent insertion/deletion mutations which occur in this population include c.187_188delAG and c.5385insC in *BRCA1* gene and c.9254_9258delATCAT along with c.3492_3493insT in *BRCA2* gene. Furthermore, investigations among 1,763 hereditary BC and ovarian cancer (OC) families have recently found that c.66_68delAG, c.5123C>A, c.1961delA, c.3770_3771delAG, and c.5152+5G>A mutations were in 45.2 % of *BRCA1* mutation carriers. In addition, c.9026_9030delATCAT, c.3264insT, and c.8978_8991del14 comprised the 43.2% of *BRCA2* alterations [67]. It was revealed that the deletion of 6 flanking exons (8–13) which also was detected in French BC patients is as a result of Alu repeat recombination in juxtaposed introns. Among all the SNPs found for *BRCA1 *and* BRCA2* genes in Spanish persons, it seems that only 9475A>G could affect structure and function of *BRCA2* protein. Another Spanish population in North America (Colorado) demonstrated the same pattern in the absence of 5382insC and 6174delT and the presence of 185AGdel Ashkenazi Jews founder mutations, that is, similar to other Spanish studies [68]. The presence of Spanish family who suffered from ovarian cancer and early onset ovarian cancer or BC is a major clue for the screening of *BRCA1 *and* BRCA2* gene mutations [40].

The general prevalence of rearrangement in *BRCA1* gene was 3% in German BC patients and their families. Such low prevalence was also already reported in Finnish and Danish BC patients [69]. However, the most common mutations of the Germany patients comprise 55% of all the mutations of *BRCA1 *and* BRCA2* genes including loss of exon 22 and exon 17 as founder deletions in *BRCA1* besides deletion of exon 13. Alu repeat slipped misspairing in this gene is responsible for these duplication/deletion aberrations. *BRCA2* gene mutations were found to be very rare in German BC families [70, 71]. Role of Alu repeat rearrangements of *BRCA1 *and* BRCA2* genes in BC pathogenesis has turned out to be more prominent in Portuguese population studies. The most prevalent mutation (1/4 of all *BRCA1 *and* BRCA2* mutations) and founder insertion are c.156-157ins Alu. It is a targeted insertion rather than random integration of 350 bps from Alu Ya5 subfamily into the exon 3 of *BRCA2* gene leading to its deletion and consequent loss of transactivation domain. It has been shown that this insertion type is responsible for about 36% of *BRCA* gene mutations in male BC families and definite identification of this insertion increases the overall detection rate of *BRCA1 *and* BRCA2* alterations up to approximately 13% [72]. Another type of insertion occurs in *BRCA1* gene through rearrangement in Alu Sp/Sq repeat family [73].

French population has shown its own specific founder *BRCA* mutations too. *BRCA1 *and* BRCA2* 2 mutations account for more than 40% of all French families affected by BC, particularly, in younger ages [74]. All of the most important mutations of this population are represented in Table 3. It was suggested that the alterations of nucleotides in spectrum of 2401–4191 in *BRCA1* gene are associated with either low risk BC or high risk ovarian cancer. Moreover, rare cases of LGRs were detected among BC patients with origin of French but not in French-Canadian [75]. Although two of the frequent mutations (6503delTT and 3398delAAAAG) take place in ovarian cancer critical region (OCCR) located in exon 11 of *BRCA2*, there is no difference in risk of ovarian cancer among the carriers of theses mutations and others which are carriers of mutations in other part of *BRCA2* gene [76]. A large deletion encompassing exons 8–13 of *BRCA1* gene and the duplication of exons 3–8 and exon 18–20 were identified in French BC families [74]. The French-Canadian BC families share a common mutation (8765delAG) in *BRCA1* gene with Jews of Yemenites origins [77]. It is interesting that except of one founder deletion in *BRCA1* gene (c.1592delT), the researchers could not find any duplication or deletion mutations in *BRCA1* and *BRCA2* genes in French origin inhabiting in Canada compared to those in France. Among all of the variants that were detected in both *BRCA1 *and* BRCA2* genes, only the c.5095>T has considerable clinical impact on *BRCA1* function [76].

The *BRCA1 *and* BRCA2* mutation studies have been performed in the Anglia, British, Scottish, Welsh, and Irish populations (Table 3). The Scottish and Irish BC patients were carriers of two Ashkenazi Jews founder mutations, 185 delAG and 5382 insC which include the most frequent *BRCA1 *and* BRCA2* mutations, respectively. Interestingly, the 4184 del4 mutation in *BRCA1* has been found in BC patients with Welsh, South Thames, Oxford, Yorkshire, Irish, and Scottish origin [78–80].

### 5.4. Eastern Europe

All the mutations regarding Eastern European population were brought in Table 4.

Alu insertion in intronic sequences was detected in Czech population in intron 3 of *BRCA1* gene made by AluSx (g.17686–17695) and AluY (g.18760–18769) repeats rearrangements and in intron 10 by means of AluJb (g.33248–33276) sequence recombination. In addition, deletion of exons 21–24 in *BRCA1* gene also was demonstrated in this population which is as a result of Alu repeats recombination. It is important in *BRCA *genes mutations screening to note that the deletion of exons 5–14 (the most prevalent rearrangement in Czech) and all of the first 17 exons of *BRCA1* gene is specific to the Czech BC patients and is also considered as founder mutations [81].

The Slovenian BC cases demonstrated the three main mutations in *BRCA1*: c.1687C>T, 5382insC, c.844_850dupTCATTAC, and c.181T>G, with rate of 56, 32, 37.3, and 30% of all BC cases respectively. Replication of the first three mentioned mutations of *BRCA1* gene in different Slovenian sample studies is relying on their consideration in mutation screening of this population [82–84]. Among the mutations detected in *BRCA2* gene, the most and the least frequent changes (c.7806-2A>G and c.3975_3978dupTGCT) were recognized in male BC cases. Additionally, c.7806-2A>G and c.5291C>G are only defined in Slovenian populations, and c.7806-2A>G and IVS16-2A> were considered as a founder mutation of this region [83, 85]. In an assay on 379 HBOC Slovenian families, c.181T>G, c.1687C>T, and c.844_850dupTCATTAC were the most frequent *BRCA1* mutations while c.7806-2A>G splicing alteration which was found in 13 families was the most *BRCA2* one [86].

In polish populace, in addition to the most frequent variants including 5382insC (exon 20), Cys61Gly (exon 5), and 185delAG (exon 2), two new insertion/deletion mutations were identified in *BRCA1 *and* BRCA2* recently (2991del5 and 6238ins2del21, resp.). 5382insC and 300T>G (Cys61Gly) were supposed as founder mutations in various polish studies [87, 88]. A new c.190T>G mutation of this population in *BRCA1* gene is associated with activation of a cryptic splice site in exon 5 and leads to truncated protein by deletion of 22 nucleotides [89]. In general, there is a high trend in *BRCA1* mutation screening among this population due to their high frequency even in BC patients without familial history [90].

In Greek territory, two major mutations were described. After 5382insC, G1738R is the most important and frequent mutation which occurs in exon 20 of *BRCA1* gene. The pathogenic effect of G1738R alteration was confirmed in many studies. It seems that G to A base pair exchange is associated with disruption of protein folding influencing the interaction of *BRCA1* with DNA damage-associated kinas BACH1 [25, 91, 92].

The incidence of *BRCA1 *and* BRCA2* gene alterations in middle and south regions are about twice that in north of Sardinia (17-18% against 7% in north). The c.1632 A>T, c.1638 A>T missense mutations and two deletions (c.3823_3826delACAA and c.4575delA) in *BRCA1*, and the c.3950_3952delTAGinsAT and c.6586 C>G in *BRCA2* gene were discovered for the first time in this population and were incorporated in BC information center (BIC). The c.8764_8765delAG and c.3950_3952delTAGinsAT del/ins mutations were considered as founder mutations in *BRCA2* gene of Sardinian population [93].

### 5.5. America

All the mutations regarding American population were represented in Table 5. However, there are some unpublished mutations acquired by next generation sequencing in Myriad Genetic Laboratories (Salt Lake City, UT) provided by BIC. In America, *BRCA1/2* mutation screening has been extended to genetic analysis of biological samples and sending reports via online genetic testing in centers as well as Myriad Genetic Laboratories which has arisen some debates regarding ethics of information obtained. However, there are several reports of various *BRCA1 *and* BRCA2* mutations found in different states of America.

In the BC families who were genotyped from Boston, 6697delTC has been shown as the most frequent mutation accompanying the three major Ashkenazi Jews insertion/deletions. Nevertheless, the same study in Washington could not find the 185AGdel [94].

In another USA study which was carried out on more than 300 BC families, the AluSx/Sx (dup 9700) and AluSx/Sp (del2352ins12) rearrangements were demonstrated in *BRCA2* genes of patients with English and Dutch in addition to German origin, respectively. Some other *BRCA1 *gene rearrangements including AluY, AluJ, AluJO, AluSg, AluSp, AluSx, AluSq, and AluSc were identified in cases with other European ancestry. All of these Alu rearrangements took place in exons unlike those occurring in introns in Nigerian BC patients screened in USA [95]. Another large 36.4 kb deletion as a result of Alu rearrangement (exons 9–19 del) was found in a Brazilian BC patient affected by Li-Fraumeni syndrome [96]. Moreover, a smaller deletion, exons 9–12 del, was identified in a Hispanic survey which is considered as Mexican founder *BRCA1* alteration with high frequency (10–12%) [97]. C61G which was formerly reported in Latvia and Belarus studies was identified in a USA survey among Caucasian women with triple negative breast cancer [98]. In another USA assay which investigated a diverse population of African American, Asian, Hispanic or none-Hispanic Ashkenazi Jews, and non-Jewish whites, the 185AGdel in different haplotype of Ashkenazi Jews was found in a woman with Irish/German origin which was never detected neither in German or Irish population [99]. In three German families, 5950delCT was identified in *BRCA2* gene as expected from previously performed German population studies [100].

Pattern of *BRCA1 *and* BRCA2* mutation was different in Southern USA studies (Table 5). The 185AGdel and 5382ins mutations have been found in Chile and Brazil assays, respectively. Study in Bahamas which is located in Central USA (a Caribbean island) showed that this population harbors the most frequency of *BRCA1* mutations (22.9%) [39, 101–104]. It was approved in a recent study on 214 Bahamian women affected by breast cancer with or without family history. 27% of them were carriers of either *BRCA1 *or* BRCA2* mutations more than any other population [105]. 

### 5.6. Asia

All the mutations regarding all Asian population were represented in Tables 6 and 7.

BC incidence is the most in Pakistan compared to other Asian populations. However, *BRCA1 *and* BRCA2* mutations play pivotal role in only 12% of all BC cases. Four truncation and frame shift in addition to one splice site mutations in *BRCA1* gene (3889delAG, 2080insA, 4284delGA, 4184del4, and IVS14-1ArG) and the missense 3337C>T mutation in *BRCA2* gene comprise the directory of founder mutations of this population. The truncated protein producing by c.4627C>A substitution is the most common alteration in *BRCA1* gene. Punjab embraces the ethnicity harboring the most rate of *BRCA1* mutations (57%) with great difference versus Muhajir groups (17%). The same situation is held for *BRCA2* mutations (33%) against multiracial women (28%) [106]. The 4627C>A, 185delAG, and 185insA mutations of *BRCA1 *which are accumulated in northern and eastern Pakistan are considered as founder alterations as 185delAG is for Pathaan, and 185insA accompanying 4627C>A is specific to Punjabi women [107].

Regardless of deletion in other populations, the LGRs which are associated with amplification were initially detected in exons 3 and 10 of *BRCA1* gene in two Malaysian women who both were affected with sporadic BC [108]. However, a multiethnic study on BC Malaysian demonstrated that the frequency of TP53 mutations is superior to *BRCA1/2* genes alterations especially in patients with positive family history which could make *BRCA1/2* mutation screening debatable [109].

The c.7480C>T mutation includes 25%–50% (in different samples) of all mutations happening in *BRCA2* and is assumed as founder and the most common alteration of *BRCA1 *and* BRCA2* genes in Korean BC women especially those who are positive for familial history and have bilateral or multifocal tumors. Among all mutations which were found in both familial and sporadic BC cases [110, 111], exon 11 was more influenced by several insertions/deletions and single base pair frame shift mutation in both *BRCA1 *and* BRCA2* gene. It is noticeable that only one LGR (c.4186-1593_4676-1465del) and in only *BRCA1* gene was reported in this population [112]. The 1627A.T (exon 10), 3972delTGAG (exon 11), and 7708C.T (exon 15) harmful mutations in *BRCA2* were replicated in multiple Korean population studies [113]. 

It is obvious that in Japanese BC patients like Korean and Cypriot ones [27], the role of *BRCA2* mutations is more frequent and important than *BRCA1*. However, among three founder Japanese alterations, the truncation mutation in codon 63 and an insertion in exon 22 are located in *BRCA1* and the third one was found in *BRCA2* gene (5802del AATT). In addition the pattern of accumulation of mutations in exon 11 of either *BRCA1 *or* BRCA2* genes mimics the Korean population [114, 115].

Several Chinese studies have relied on the three major mutations in *BRCA1* gene including 3478del5, 5589del8, and 1100delAT, with the first being only annotated in Chinese women [116, 117]. It was suggested that both of them are considered as founders of north China. These mutations have shown correlation only with familial not sporadic BC [118, 119]. Another founder mutation of *BRCA1* has been introduced which is specific to BC patients from Hong Kong and southern China (Table 6) [120, 121].

It is surprising that the 185delAG deletion takes place in high incidence of 16.3% in Indian population near the frequency of 18% in Ashkenazi Jews. In addition, the role of *BRCA2* mutations is meaningless in Indian BC genetics especially in earlier ages [122–125]. In this way, more focus on *BRCA1* gene mutations led to uncovering two 1014delGT and 3889delAG premature stop codon deletions in addition to 5382insC in BC patients from North-East and Eastern India, respectively [126, 127].

In a recent mutation screening of *BRCA1* gene on Russian 7920 normal and 570 BC patients, each of 185delAG and 4153delA mutations were identified in only one BC case. However, 5382insC and 300 T>G were recognized in both normal and BC groups [128]. 5382insC and 4153delA had been identified in three previous Russian studies while 5382insC had shown more frequency than 1100delC mutation of *CHEK2* among Siberian BC patients compared to healthy controls [129–131].

Based on piecewise Weibull model and in a kin-cohort analysis, we have found that the *BRCA1/2* mutations penetrance seems to be lower among Iranian breast cancer families as it was reported 31.9% and 46.2% for women aged lower and more than 50 years old, respectively [132]. In the Iranian *BRCA1* mutation analysis, DNA samples of 80 early onsets BC patients were undergone PCR sequencing. The novel 1534G>A substitution was detected in exon 16 of *BRCA1* in 38.6 of BC patients as well as 52.8% of healthy controls. They could find another mutation in exon 15 of *BRCA1* besides eight variants in which Pro871Lue and Glu1038Gly were identified in the same haplotypes. Moreover, it was shown that Leu871 was significantly more in controls versus patients (*P* < 0.01) which is inconsistent with previous studies implying the higher frequency amongst BC patients [133]. Yassaee and his coworkers found a polymorphism (duplication of 12 base pairs in IVS20+48) in exon 20 of *BRCA1* gene along with 6261insGT inside the *BRCA2* gene in a 27-years-old BC patient. One patient showed IVS16-14T>C within exon 17 of *BRCA2* close to the splice site which has been previously reported in British population. They have reported the 5382insC in 19% of their familial BC patients, with possible role of it as a founder mutation of Iranian nonfamilial BC cases [134]. The novel G2031T base pair exchange was determined in *BRCA1* gene through investigation of exons 2, 20, and 11 in an Iranian family who suffered from hereditary breast cancer and ovarian cancer syndrome [135]. We performed mutation screening of all coding and 3′ and 5′ UTR sequences of *BRCA1 *and* BRCA2* genes on ten high risk breast cancer families. A novel *BRCA2* mutation c.4415 4418delAGAA was discovered and insertion c.6033_6034insGT was also found which could lead to premature termination of translation at the codons 1477 and 2040, respectively. In *BRCA1* gene, the IVS17-53C>T and g.381-389del9ins29 of 3′ UTR were found. The latter was detected in two families, in one of those two affected sisters and their healthy mother were negative for g.381-389del9ins29 of 3′ UTR alteration [34]; such familial screening has a positive clinical impact on the quality of life of proband's relatives and could be considered as a personalized management. By considering the specific screening program in our study, the 185delAG was found in one BC patient and her sister was affected with meningioma in age of 46 and 35 yrs, respectively, and also in another proband from separate family. We could identify neither 5382insC nor 6174delT in the studied population [136]. The same study was conducted by Fattahi and his colleagues in which no Ashkenazi Jews alteration was found [137]. However, the 5382insC in 20% of their familial BC patients was reported [138]. Some other novel mutations were detected in *BRCA1* (p.Glu1735 p.Gly1140Ser, p.Ile26Val, p.Leu1418X, p.Glu23Gln, p.Leu3X, p.Asn1403His, p.Lys581X, p.Pro938Arg, p.Thr77Arg, p.Arg7Cys, p.Ser177Thr, IVS7+83(TT), IVS8-70(-CATT), IVS2+9(-GC), IVS1-20(-GA), IVS1-8(-AG), IVS2+24(AG), IVS5-8 (A-G), and IVS2(35–39)TTCCTATGAT) and in *BRCA2* (p.Glu1391Gly, 1994_1995 (Ins A), and IVS6-70-T>G) genes recently [139]. 

An Iranian BC patient who was also none-Ashkenazi Jewish has demonstrated a founder mutation in *BRCA1* gene (Tyr978X) as well as Iraqi, Afghani, and Canadian BC patients from Israel [140]. It was replicated in a later study carried out in Israel among non-Ashkenazi Asian BC patients [141]. This mutation was not previously detected in the same non-Jewish Iranian and Iraqi populations [142].

### 5.7. Africa

All the mutations regarding the USA African population have been compiled in Table 8.

In all the few African population assays which we have reviewed, only Egyptian women demonstrated founder mutations in both *BRCA1 *and* BRCA2* genes. However, African triple negative BC patients from USA showed 943ins10 mutation of *BRCA1* as founder of West Africa [98]. The deletion of exon 21 in *BRCA1* gene was identified in a woman who was one of the 352 participants Nigerian *BRCA* screening. This deletion is a result of unequal crossing-over of AluSg and AluY repeat from introns 20 and 21, respectively [14]. Two major Ashkenazi Jews mutations of *BRCA1* gene, 185AGdel and 5382insC, were identified in Egyptian and Tunisian women, respectively. The c.798_799delTT alteration of *BRCA1* was shown in Tunisian BC cases from Algeria [143]. However, the 185AGdel founder alteration was found in two independent screening tests of Jewish and non-Jewish Morocco population [144]. The 5999del4 mutation was found to be a founder *BRCA2* mutation of Western Cape Province which had been found previously in Dutch population without any evidence of common ancestry for these two populations [145]. Moreover, in a recent study, c.798_799delTT was introduced as a non-Jewish founder *BRCA1* gene identified in Tunisian and Algerian BC patients and their families inhabitant in Northwest Africa (Morocco) [146].

### 5.8. Other Continents and Islands

Another population that is famous to have founder mutation is Greenland. In addition to 234T>G and 249T>A founder mutations which were originated from Danish population, the 4803delCC, p.Cys44Phe p.Cys44Tyr (c.131G>A), and p.Cys44Ala should be considered in mutation screening of *BRCA1* in Greenlandic women [147]. 

Although in Australia continent the role of insertion/deletion mutations in *BRCA1 *and* BRCA2* genes is weaker, some single nucleotide polymorphisms (SNP) showed significant association with BC risk which was discussed in polymorphism section. No important, founder and frequent *BRCA1 *and* BRCA2* mutation was yet reported in Australian studies [148].

## 6. Clinical-Pathological Correlation with *BRCA1* and *BRCA2* Mutations

The effect of *BRCA1 *and* BRCA2* mutations on histological and pathological state, stage, and grade of BC, especially the involvement of lymph nodes, is determining factor of BCs prognosis and survival rate. In general, the carriers of *BRCA1 *and* BRCA2* show higher tumor stage, grade and ER negative tumors, and more metastasis to neighbor vessels relative to those who harbor other gene mutations [149]. It was in agreement with approximately all of ethnics and population especially Asian BC families compared to non-*BRCA1 *and* -BRCA2* mutations, and also among the *BRCA1 *and* BRCA2* alterations, *BRCA1* mutations are associated with higher tumor grade, P53 mutations and higher basal cell markers including cytokeratin [150, 151] and P-cadherin expression while underexpression of E-cadherin in triple negative BC cases of younger ages (estrogen, progesterone, and Her2 receptors) and eventually weaker prognosis [116, 152]. In addition, we have reported that significant correlation was found between expression of *BRCA1* gene and degree of tumor as upregulated gene expression was related to the higher tumor grade [153].

Therefore, it seems that hormones disturbances have no role in the development of BC in *BRCA1 *and* BRCA2* mutations carriers. Moreover, *BRCA1* mutations frequently occur in medullary especially atypical BC with higher mitotic index (>50/10HPF) versus tubular and intraductal ones which are more seen in *BRCA2* mutations. Another tumor morphology pattern of *BRCA1* carriers comprise of pushing margins which avoid of tubular formation, trabecular, syncytial and circumscribed growth pattern and necrosis [154]. However, a recent study on American population has demonstrated higher significant survival (relapse-free survival or RFS) in both *BRCA1 *and* BRCA2* carriers compared to non-*BRCA* BC cases [155]. It was shown that some LGRs in *BRCA1* gene including exons 17 and 20 deletions were associated with previously defined tumor morphology [156].

It is noticeable that the French *BRCA1 *and* BRCA2* mutation carriers are usually negative for lymph nodes involvement and contralateral form of BC. However they have shown higher tumor stage and lower rate of ductal in situ carcinoma [157].

## 7. Discussion

The most frequent *BRCA1* gene mutation is 5382insC (c.5266dupC) which was found in roughly all of the populations. The maximum likelihood method considering any mutation or crossing-over occurrence has shown that this insertion at first came from Scandinavia probably Denmark as it comprises the founder mutation in Danish population around 200 AC. However, Russia is another candidate for occupying the primary origin of it and after that it was disseminated to other areas including Ashkenazi Jews. It was also proposed that 5382insC has entered into the Ashkenazi Jewish through affecting Polish population about 400 yrs earlier. 5382insC is the most important and prevalent *BRCA1 *mutation in European countries even though; Asian and American BC individuals rarely demonstrate it [158]. The second globally frequent *BRCA1* mutation is 185AGdel in exon 2 and was described in all the ethnics including Asia, America, Africa, and European populations. Since, it was replicated in various populations with Arabic ethnics including Syria, Iraq, and Yemen, considering this deletion in screening high risk families with Arab ethnics may be helpful. The third most frequent *BRCA1* alteration is 300T>G which has mainly determined in East European populations in addition to Germany. It is interesting that African American population from USA have demonstrated 300T>G as a recurrent *BRCA1* mutation [99]. Shedding light on the common ancestry of this mutation in two unrelated African American and Eastern European populations may be helpful for finding a logical way to consider it in screening programs of other related populations. Except of the most recurrent mutations which were described, approximately all of the populations, even among the same ethnics, show different *BRCA1 *and* BRCA2* mutation may patterns. Although with inventing the new mutation screening methods like next generation sequencing knowing the most frequent *BRCA1 *and* BRCA2* mutations not be required, some countries especially the developing countries may take some advantages. In addition, if we have the most recurrent *BRCA1 *and* BRCA2* mutations of our ethnics or population, the diagnosis and treatment of BC patients and the process of followup of their family get faster and better. 

Taken together, paying attention to the frequent mutations facilitates analysis in high risk members of BC families in all populations worldwide.

## Figures and Tables

**Table 1:** The most important and frequent polymorphisms of *BRCA1* and *BRCA2* genes around the world.

Gene	Type of polymorphism	
	Unclassified polymorphisms	Missense polymorphisms
*BRCA1 *		E1038G, P871L, K1183R, S1613G, M1652I, D1778G [19], S1436S, L771L, E1038G [23], A1708E [33]
	IVS7+36T>C, IVS7+38T>C, IVS7+41C>T, IVS7+49del15 [23], (IVS16−68G>A, IVS16−92G>A, IVS18+65G>A [23]	c.2196G>A, c.3232A>G, c.3667A>G, c.4956A>G, c.5075G>A [23]
	S1040N [27], g.5075−53C>T, g.*381_389del9ins29 [31]	Q944E, S919P [24], c.4185+3A>G [27], c.302−3C>G, c.4185G>A and c.4675+1G>A [26], IVS18+1 G>T, 5632T>A (V1838E) [30], Y179 in association with F486L [29], Q356R and S1512I [36], 3232A>G [37], L1198W and R1347G [35], c.9023A/C [36], 4817A>G [38]

*BRCA2 *	IVS16−14T>C [35]	c.475G>A, c.7007G>A, c.476−2>G; c.7007G>A; c.8755−1G>A; c.9117+2T>A and c.9118−2A>G [28], 1342A>C [37], S1832P, T2766I, N2781I, and K2860T [35], c.72A>T [36], K3083E or 9475A>G [40]

**Table 2:** *BRCA1* and *BRCA2* gene mutations in Northern Europe.

Country	*BRCA1 *mutations	*BRCA2* mutations	Founder mutations
Finland	c.5095C>T [3],4216-2nt A–>G 5370C>T [159]	4088insA, c.68-80insT, c.793+34T>G 999del5 6503delTT [159]	4216-2ntA>G 5370C>T 999del5 6503delTT [159]
Sweden	3172ins5, 2594delC 1806C>T, 1201del11 [47, 48]	4486delG [47, 48]	3172ins5, 2594delC 1806C>T, 1201del11 4486delG [47, 48]
Denmark	3172ins5 and 1201del11 1675delA and 1135insC [38, 49]	2594delC, 5382insC 3829delT and Q563X 3438G>T, 1675delA, 999del5 [38, 49]	Iceland/Denmark (999del5) Norway/Denmark (1675delA and 1135insC) Swedish/Danish founder (3172ins5 and 1201del11, 3438G>T, 1675delA) Danish specific: 2594delC, 5382insC, 3829delT, and Q563X [38, 49]
Norway	1135insA, 1675delA, 816delGT, 3203del11, and 3347delAG [50, 51]	—	—
Iceland	G5193A [54]	999del5 [54]	999del5, G5193A [54]
Netherland	Exon 13 and 22 del 2804delAA IVS2011G>A, IVS21-36del510 5382insC, 1411insT, 2138delA 2312del5, 2457C4T, 185insA, 185delAG [59]	6503delTT, 8295T4A, 9900insA, 5579insA, 7647delTG [59]	5579insA, exon 13 del [59], 2804delA [160]

**Table 3:** *BRCA1 *and *BRCA2* gene mutations in Southern, Central and Western Europe.

Country	*BRCA1* mutations	*BRCA2* mutations	Founder mutations
Switzerland	G4956A [161]	7253delAA [161]	—
Britain	4184del4 [78–80], 185DelAG, intron 5 splice, 5382 Ins C [80, 162], C4446T, 3875del4, 2800delAA [27], 2080delA [80], 2594delC [79]	6503delTT, 9303ins31 [80]	2594delC [welsh founder [79], 2800delAA [163], 1294del40 [162] 2157delG [76]
Germany	655A>G [164], 4282delAG [165, 166], 300T>G, 5382insC, 4184del4bp [100], c.3700_3704del5( exon 17 and 22 del Exon 13dup [71]	3034del4bp, 5910C3G, 6676insTA [100]	2457C3T, 5382insC, 300T3G [100]
France	French-Canadian: 4446C>T, 2953delGTAinsC, R1443X, 3875delGTCT [76, 167–169] French: 3600del11, 4184del4, G1710X [170] Exons 8–13 del Exons 3–8 and 18–20 dup [170, 171]	French-Canadian: 6085G>T, 8765delAG, 3398delAAAAG, 6503delTT [76, 168, 169]	3398delAAAAG [73]
Italy	c.3228_3229delAG, c.3285delA, c.1377_1378insA c.5062_5064delTGT Exons 17, 9–19, 18–19, 1a-2, 16–20 del 4843delC [59]	1499insA [21], 7525_7526insT, 6174delT [20] c.289G>T, c.2950G>T c.7963C>T and c.8878C>T [61, 62]	c.1377_1378insA and c.5062_5064delTGT Exon 17 del, 4843delC [20], 1499insA [21, 172]
Belgium	IVS5+3A>G [173], E1221X, 2478–2479insG [174]	IVS6*þ*1G4A, 6503-6504delTT, 9132delC [174]	IVS5+3A>G [173]
Spain	330A>G, c.187_188delAG, c.5385insC, c.5242C>A [68] c.66_68delAG, c.5123C>A, c.1961delA, c.3770_3771delAG, and c.5152+5G>A [67] Exons 3–5 del [66]	9254del5, c.9254_9258delATCAT, c.3492_3493insT, 9475A>G [68] c.9026_9030delATCAT, c.3264insT and c.8978_8991del14 [67]	330A>G, 9254del5 6857_6858del [65] Exons 3–5 del [66]
Portugal	—	c.156_157insAlu [70]	c.156_157insAlu [70]
Poland	4153delA, 5382insC, 300T>G, 185delAG, 3819del5, c.190T>C, 2991del5 [87, 88],C5370T, 3875del4 [90]	6238ins2del21 10323delCins11 8876delC [87]	5382insC and 300T>G [87, 88]

**Table 4:** *BRCA1* and* BRCA2  *gene mutations in Eastern Europe.

Country	*BRCA1* mutations	*BRCA2* mutations	Founder mutations
Czech	5382insC, c.3819_3823del5, 300T>G [28], AluSx ins (g.17686-17695), AluY ins (g.18760-18769) AluJb ins (g.33248-33276) Exons 21–24 del Exons 5–14 del, exons 1–17 del, c.3700_3704del5 [175]	8138_8142del5, c.8765_8766delAG [28], exons 21–24 del [175]	300T>G, c.8765_8766delAG 8138_8142del5 [28], exons 5–14 del Exons 1–17 del 5382insC [175]
Slovakia	5382insC [176, 177], 300T>G, 185delAG, c.843_846del4 [177, 178], 300T>G, c.3700_3704del5, c.4243delG [178]	c.6589delA [178, 179]	c.6589delA [178, 179]
Romani	5382insC, 300T>G, 461delTC [41]	4817A>G, 8477delAGA [41]	—
Greek (South eastern Europe)	5382insC and G1738R [92] G5331A, 3819delGTAAA [180]	8984delG [35], G4X and 3783del10 [92]	5382insC and G1738R [92], 3782del10, 4512insT [180]
Slovenia	c.1193C>A [86] c.181T>G, c.1687C>T, 5382insC, c.844_850dupTCATTAC [85] 300T>G, 1806C>T, IVS16−2A>G [83] c.116G>A, c.844_850dupTCATTAC, c.1687C>T, 300T>G [82]	c.5101C>T, c.5433_5436delGGAA [86] c.7806−2A>G, c.5291C>G, c.3975_3978dupTGCT [85] IVS16−2A>G [82]	5382insC, c.7806−2A>G IVS16−2A>G [83]
Austria	300T>G, 2795del4, C1806T, 5382insC [181]	—	300T>G [181]
Croatia	c.3318C>A and c.4790C>A [181]	c.3318C>A, c.4790C>A [181]	—
Latvia	4153delA, 5382insC, C61G [182, 183]	—	5382insC [182, 183]
Hungary	5382insC, 300T>G, 185delAG [184, 185]	9326insA and 6174delT [184]	5382insC, 185delAG [184, 185]
Yugoslavia	5382insC, 185delAG, 3447del4 [186]	—	5382insC [186]
Belarus	4153delA, 5382insC, C61G [187]	—	4153delA and 5382insC [187]
Cyprus	5429delG [35], 3232A.G, 4956A.G [27]	8984delG [35], 1913T>A [36], 1342C>A, 3199A>G, 1093A>C [27]	8984delG [36]

**Table 5:** *BRCA1* and *BRCA2* gene mutations in America.

Country	*BRCA1* mutations	*BRCA2* mutations	Founder mutations
Cuba	c.5231delT [188]	c.3394C>T, c.7697T>C [188]	—
Costa Rica	C3522T [189]	5531delTT, C5507G and 6174delT [189]	—
Chile	185AGdel [39, 104]	c.5373_5376 del GTAT, c.373G>T [104]	—
Brazil	5382insC [101, 102, 190]	S2219X, C1290Y [101], 6633del5 [102]	—
Colombia (Hispanic and Colombia)	3450delCAAG [33, 191] and A1708E (polymorphism) [33] Exons 9–12 del [97]	3034delACAA [33]	3450delCAAG [191] Exons 9–12 del (Mexican founder) [97]
Bahamas	IVS13+1G>A, 4730insG, T5443G, IVS16+6T>C, 943ins10, 185delAG [103]	818delA, exons 8-9 del [105]	818delA [105]
Venezuela	c.951_952insA, c.1129_1135insA, c.4603G>T and IVS20+1G>A [192]	c.3036_3039delACAA, c.6024_6025_delTA, c.2732_2733insA and c.3870_3873delG [192]	—
Puerto Rico	Exons 1-2 del [193]	4150G>T, 6027del4 [193]	—
Mexico	c.3124_3133delAGCAATATTA c.2805_2808delAGAT [194]	c.5114_5117delTAAA c.2639_2640delTG [194]	—

**Table 6:** *BRCA1* and *BRCA2* gene mutations in Northern and Eastern Asia.

Country	*BRCA1* mutations	*BRCA2* mutations	Founder mutations
Turkey	5382insC, 5622C>T [22, 195, 196]	6880 insG and 3034 del AAAC [197]	Maybe 5382insC [195]
Russia	5382insC [130, 131], 4153delA [129] 185delAG [128]	695insT, 1528del4, 9318del4, S1099X [131]	5382insC [131]
Japan	c.307T>A [115]	5802delAATT, 8732C>A, c.2835C>A [114, 115]	c.188T>A, c.2800C>T [110] c.2835C>A, c.307T>A, 5802delAATT [114, 115]
Korea	509C>A, c.2333delC, c.4065_4068delTCAA 3746_3747insA (c.3627_3628insA ), 5199G>T (c.5080G>T) [110, 198]	c.7480C>T, 1627A.T 3972delTGAG, 7708C.T [110, 111, 198, 199]	c.7480C>T [110, 111, 199]
China	3478del5, 5589del8, 1100delAT, 2778G>A, 3552C>T [117, 118], exon 10 dup, 5,273G>A [108, 117] c.470_471delCT, c.3342_3345delAGAA, c.5406+1_5406+3delGTA and c.981_982delAT [120]	7883delTTAA [200] c.2808_2811delACAA, c.3109C>T, c.7436_7805del370, and c.9097_9098insA [120]	Hong Kong: 5589del8, 1100delAT [121] Southern China: c.3109C>T, c.3109C>T, c.7436_7805del370, c.981_982delAT, c.7436_7805del370, and c.9097_9098insA [120]
	2845A>T [201], 3300delA, T320G [202] c.5191C>A [203]	2670delC, 3073delT, and 6696-7delTC [204]	3300delA, T320G

**Table 7:** *BRCA1* and *BRCA2* gene mutations in Southern and Western Asia.

Country	*BRCA1* mutations	*BRCA2* mutations	Founder mutations
Singapore	Exon 13 dup, 13–15 del [205], c.2845insA [206]	Exons 4–11 dup [205]	c.2845insA [206]
Malaysia	c.2845insA [206], 4427T>C, 2846insA, 2201C>T and 4956A>G (79%), 3668A>G [207], 2731C>T, 3232A.G, 3667A.G [24], exon 3 dup [108]	4859delA, 4265delCT [208], 1342C.A [24] 490 delCT	c.2845insA [206]
Pakistan	4627C>A (22%) [107], 4184del4 (15%), 185delAG and 2080insA and IVS14−1G>A (11%), 2041insA and 4284delAG (8%), 3889delAG and 2388delG (7%) [106]	3337C>T (50%), 5057delTG (50%) [106]	4627C>A, 185delAG, 185insA [107]
Iran	g.-1075C>G, g.-235A>G g.-134T>C, g.442−34C>T, g.548–58delT c.2077G>A, c.2082C>T c.2311T>C, c.2612C>T c.3113A>G, c.3119G>A c.3548A>G, c.4308T>C c.4837A>G, g.4987−68A>G, g.4987−92A>G g.5075−53C>T, g.5152+66G>A g.381_389del9ins29 g. 421G>T, g. 1286C>T [34] IVS16−92A>G, IVS16−68A>G, 4837A>G, IVS18+65G>A [133], Tyr978X,	g.-1235G>A, g.-26G>A g.681+56C>T, c.865A>C c.1114A>C, c.1365A>G c.2229T>C, c.2971A>G c.3396A>G, c.3516G>A c.3807T>C c.4415_4418delAGAA c.5529A>C, c.6033_6034insGT c.7242A>G, g.7435+53C>T g.7806−14T>C g.8755−66T>C [34] c.4415-4418delAGAA and c.6033insGT [134] c.5576_5579delTTAA c.9485−1G>A [209]	—
Lebanon	IVS17−53C>T and g.381-389del9ins29 [134], 5382insC [138] G2031T [135]	—	—
India	185delAG [122, 123], 2983C>A, 3450delCAAG [123, 124], c.3548A>G, c.-26G>A, c.317-54C>G [125], 5341T>G, 5364C>G, 5379 G>T [210] 1014DelGT and 3889DelAG [126] 5382insC [127]	—	185delAG [122]
Sri Lanka	c.3086delT, c.5404delG, c.856T>G, IVS17−2A>T [23]	—	—
Philippines	5454delC [208]	4265delCT and 4859delA [208]	5454delC, 4265delCT, 4859delA [208]
Indonesia (in South eastern Asia)	—	6775G>T, p.Glu2183X, c.2699_2704delTAAATG [211]	c.2699_2704delTAAATG [211]
Vietnam	185insA [212]	4706delAAAG [212]	—
Thailand	3300delA [202]	—	—
Israel (in Western Asia)	185delAG, Tyr978X, A1708E, 981delAT, C61G [141]	R2336P, IVS2+1G>A, 8765delAG [141]	—

**Table 8:** *BRCA1* and *BRCA2* gene mutations in Africa.

Country	*BRCA1* mutations	*BRCA2* mutations	Founder mutations
Nigeria	Exon 21 del (c.5277+480_5332+672del), intron 20 (AluSg), intron 21 (AluY) [14]	—	—
Egypt	185 del AG, 5454 del C, 4446C>T, 738C>A [213]	999 del 5 [213]	999 del 5, 185 del AG, 5454 del C [213]
Tunisia	330 dupA (novel), 4160 delAG, the 2789 delG, 5385 insC [214], c.4041delAG, c.2551delG and c.5266dupC, c.798_799delTT [215]	1537 del4 and 5909 insA [214], c.211dupA [215]	—
Algeria	c.46_74del29, c.798_799delTT [143]	—	—
Morocco	c.1016dupA, c.798_799delTT, c.5095C>T, c.4942A>T, c.2805delA/2924delA [146]	c.3381delT/3609delT, c.7110delA/7338delA, c.7235insG/7463insG [216]	c.798_799delTT [146]
Western cape of South Africa	c.1504_1508del [145]	c.2826_2829del, c.6447_6448dup, c.5771_5774del, 5999del4 [145]	5999del4 [145]

## Abbreviations

ATM: Ataxia telangiectasia mutated
BASC: *BRCA1*-associated genome surveillance complex
BC: Breast cancer
BIC: BC information center
BRCT: *BRCA1* C Terminus
DBD: Double strand break domain
DNA: Deoxy nucleic acid
ER: Estrogen receptor
LGRs: Large genomic rearrangements
OB1–3: Oligonucleotide-binding 1–3
OCCR: Ovarian cancer cluster region
NLS: Nuclear localization signal
PJS: Peutz-Jeghers syndrome
PR: Progestrone receptor
SNP: Single nucleotide polymorphisms.
